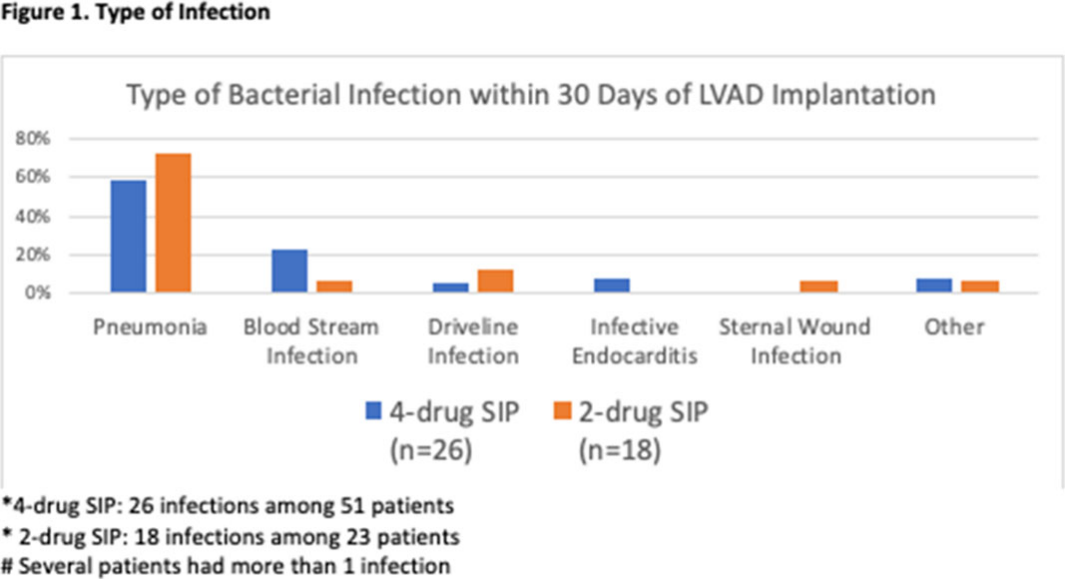# The Impact of Narrowing Perioperative Antibiotic Prophylaxis for Left-Ventricular-Assist Device Implantation

**DOI:** 10.1017/ash.2021.153

**Published:** 2021-07-29

**Authors:** Lauren Allen, Rachel Bartash, Kelsie Cowman, Yi Guo, Grace Minamoto, Snehal Patel, Sasha Vukelic, Daryl Nnani, Daphenie Fauvel

## Abstract

**Background:** Left-ventricular-assist device (LVAD)–related infections occur in 20%–40% of LVAD recipients and may result in up to 10% of LVAD-related deaths. Optimal surgical infection prophylaxis for LVAD implantation is not well defined. Our institution historically used a 4-drug surgical infection prophylaxis regimen of fluconazole, ciprofloxacin, rifampin, and vancomycin as recommended by the device manufacturer. In January 2020, a 2-drug surgical infection prophylaxis regimen of vancomycin and cefazolin was implemented to reduce broad-spectrum antibiotic use while preserving gram-positive coverage. The primary objective of this study was to compare LVAD-associated infection rates before and after changing surgical infection prophylaxis. **Methods:** A retrospective review of patients who underwent LVAD implantation between January 2018 and January 1, 2021, was performed. Definitions of LVAD-associated infections and non-LVAD infections were based on the International Society for Heart and Lung Transplantation guidelines. Infection rates at 2 weeks and 30 days after implantation and 30-day mortality were compared between the 4-drug surgical infection prophylaxis regimen (January 2018–December 2019) and the 2-drug regimen (January 2020–January 2021). Additional data collected included demographics, cause of cardiomyopathy, type of infection, and causative organism. **Results:** In total, 51 patients were in the 4-drug surgical infection prophylaxis group and 23 patients were in the 2-drug surgical infection prophylaxis group. Baseline characteristics between the groups were similar. The cause of cardiomyopathy in both groups was predominantly nonischemic (67% vs 70%, = .81), and most patients received a Heartmate III device (84% vs 100%, *P* = .06). There was no statistical difference between infection rates in the 4-drug and 2-drug prophylaxis groups at 2 weeks or 30 days (Table 1). The 30-day mortality rate was 4% in the 4-drug group versus 13% in the 2-drug group (*P* = .17). No deaths were due to infections. Gram-negative and fungal LVAD–associated infections were rare: 4% versus 4% (*P* = .99) for gram-negative infections and 2% versus 0% (*P* = .99) for fungal infections. The most commonly isolated organisms were *Staphylococcus aureus* and coagulase-negative *Staphylococcus* in both groups. Pneumonia was the most common infection in both groups (Figure 1). **Conclusions:** We did not observe a significant increase in infection or mortality with narrowing of perioperative antibiotics. However, these results should be interpreted cautiously given the small sample size. Larger studies are needed to confirm these findings.

**Funding:** No

**Disclosures:** None

**Table 1.**
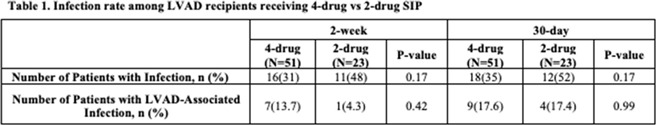

**Figure 1.**